# Understanding and designing photothermal responses in complex layered systems

**DOI:** 10.1038/s41598-025-11585-8

**Published:** 2025-07-18

**Authors:** Yide Zhang, Nelson G. C. Astrath, Gustavo V. B. Lukasievicz, Artem S. Vorobev, Liam O’Faolain, Georg Ramer, Bernhard Lendl

**Affiliations:** 1 Institute of Chemical Technologies and Analytics, TU Wien, 1060 Vienna, Austria; 2 Centre for Advanced Photonics and Process Analysis, Munster Technological University, Cork, T12P928 Ireland; 3 Tyndall National Institute, Cork, T12R5CP Ireland; 4 Department of Physics, Universidade Estadual de Maringá, Maringá, PR 87020-900 Brazil; 5 Department of Physics, Universidade Tecnológica Federal do Paraná, Medianeira, PR 85722-332 Brazil; 6 Christian Doppler Laboratory for Advanced Mid-Infrared Laser Spectroscopy in (Bio-)process Analytics, TU Wien, 1060 Vienna, Austria

**Keywords:** Optical techniques, Optical spectroscopy

## Abstract

Understanding heat transport and thermoelastic behavior in layered nanostructures is critical for designing advanced materials and devices. Here, we present a photothermal mirror-infrared (PTM-IR) spectroscopy approach that enables depth-sensitive, non-contact characterization of thermal dynamics in multilayer thin films. Using a trilayer polymer system composed of poly(methyl methacrylate) (PMMA) and SU-8 on a $\hbox {CaF}_2$ substrate, we extract layer-specific optical absorption coefficients and probe the time-resolved temperature and surface displacement evolution. We introduce a new one-dimensional (1D) Green’s function framework that provides intuitive physical insight into the temporal evolution of photothermal signals in layered structures, revealing the roles of substrate interactions and interface effects. Experimental PTM-IR signals are in excellent agreement with both a two-dimensional (2D) axisymmetric space dimension finite element model and our analytical framework, validating our interpretation of the transient thermal and mechanical responses. We show that the thermal rise time is significantly shorter than the thermoelastic relaxation time and that both the temperature and surface displacement scale linearly with the absorption layer (SU-8) thickness. These results establish PTM-IR as a powerful tool for in situ analysis of multilayer systems, with applications ranging from thermal metrology to photonic and quantum materials.

## Introduction

Measuring thermal dynamics is essential for understanding energy transport^1^, evaluating thermal properties^2,3^, and characterizing these properties at both the microscale and nanoscale^4,5^. In particular, understanding the photothermal response in complex layered systems is crucial for accurately interpreting thermal measurements^6,7^, extracting depth-resolved material parameters^8^, and guiding the design of advanced devices with tailored thermal behavior^9^.

Photothermal techniques are particularly suited to this task due to their non-contact, time-resolved, and spectroscopically selective nature. Among these, photothermal mirror-infrared (PTM-IR) spectroscopy has emerged as a powerful method that leverages mid-IR vibrational resonances to achieve layer-specific excitation and thermal probing, enabling insight into subsurface thermal and mechanical behavior^10^.

Among these complex systems, multilayer thin-film materials stand out as foundational components of modern science and technology. They enable key functionalities in optics, photonics, microelectronics, and energy harvesting^11–14^. By carefully selecting and structuring different materials, it is possible to enhance performance and realize behaviors that are difficult to achieve with homogeneous materials. Furthermore, the ability to tailor the optical, mechanical, and spectroscopic properties of individual layers makes these systems indispensable in applications such as coatings, sensors, optoelectronic components, and nanophotonic devices^15–19^. However, in contrast to single-layer structures–whose photothermal behavior has been investigated in our previous work^10^–multilayers introduce new complexities due to thermal coupling at internal interfaces and the interplay of spectrally overlapping absorbers.

However, quantifying photothermal contribution in such layered structures remains a significant challenge. The photothermal signal often represents a convolution of multiple layer-specific contributions, complicating interpretation. In addition, the presence of surrounding layers and substrate effects can substantially alter the time-resolved thermal response, making it difficult to isolate the intrinsic behavior of individual layers.

These challenges are especially relevant in the mid-IR regime, where excitation occurs via direct molecular vibrational absorption, yielding stronger and more localized heat generation compared to visible or near-infrared excitation. This differs from visible/NIR photothermal techniques that rely on electronic absorption and often exhibit deeper optical penetration and lower spectral selectivity. Additionally, PTM-IR employs mid-IR EC-QCLs for excitation and visible probe lasers for detecting nanometric surface displacement, a contrast from traditional thermoreflectance techniques based on reflectivity changes.

These experimental challenges are mirrored by theoretical complexities. Accurately modeling light-induced temperature changes and thermoelastic displacements in layered films is nontrivial^4,20,21^. In such systems, the thermal coupling between each layer and its environment – including the substrate and surrounding medium — must be considered, as it results in a complex spatial temperature distribution. This thermal landscape governs temperature-driven phenomena, including the thermoelastic response and spectroscopic behavior of the system.

To address these challenges and gain insight into the underlying dynamics, we applied photothermal mirror-infrared (PTM-IR) spectroscopy^10^ using a pump–probe approach to investigate the time-domain thermal expansion dynamics of a three-layer polymer system composed of poly(methyl methacrylate) (PMMA), SU-8, and PMMA, spin-coated onto a 2 mm thick $\hbox {CaF}_2$ substrate.

To characterize the multilayer structure, confocal Raman microscopy was used to determine layer thickness and depth distribution, while PTM-IR spectroscopy was employed to probe the spectroscopic features of the system. To analyze the time-dependent evolution of temperature and thermoelastic responses in the individual layers and substrate, we developed both a two-dimensional (2D) axisymmetric space dimension finite element analysis (FEA) model and a one-dimensional (1D) analytical model based on a Green’s function approach. Transient photothermal signals acquired via PTM-IR spectroscopy were compared against FEA simulation results.

The experimental data showed excellent agreement with FEA predictions, allowing us to quantitatively determine the optical absorption coefficients of SU-8 and PMMA. In addition, we investigated the power dependence of the photothermal signal, and the theoretical model accurately described the resulting temperature changes and surface displacements, taking into account heat transfer to the surrounding air and substrate. The influence of SU-8 layer thickness on the thermal and mechanical responses was also systematically analyzed. The analytical model showed strong alignment with the FEA simulations, providing insights into the thermoelastic displacements originating from both the polymer layers and the substrate. These results highlight the significant contribution of the substrate in PTM-IR measurements. Overall, the PTM-IR approach presented here offers a powerful tool for in situ, remote, and non-destructive characterization of thin films on non-absorbing substrates.

## Results

### Spectroscopic and transient analysis

**Fig. 1:**
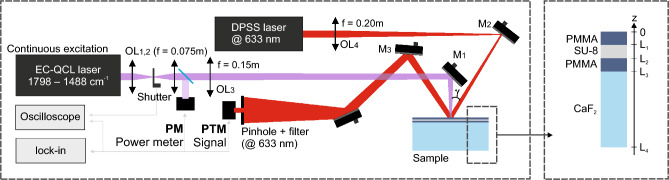
Schematic diagram of the experimental photothermal mirror-infrared (PTM-IR) setup. A tunable continuous wave (cw) mid-IR excitation laser beam is focused onto the sample surface. A visible cw probe laser is aligned along the same axis as the excitation beam to detect the focusing effects created by the excitation on the sample surface. The change in intensity at the center of the probe beam, following reflection by the sample, is detected using a pinhole-laser line filter-photodetector assembly in the far-field. Details are given in the Methods section.

Photothermal mirror-infrared (PTM-IR) spectroscopy is a non-destructive pump and probe method. The basic configuration of the photothermal mirror experimental setup is shown schematically in Fig. 1. A tunable external cavity quantum cascade laser (EC-QCL), 1798 to $1488\hbox { cm}^{-1}$, is arranged almost collinear to the visible probe laser beam to excite the sample. The effects that follow laser excitation generate a temperature change and consequent surface displacement of the layered sample. The intensity variation of the center of the reflected probe beam after a pinhole-laser line filter-photodetector assembly is detected in the far field. A digital oscilloscope was used for the transient analysis, and a lock-in amplifier was used for the spectroscopic measurements. A detailed description of the PTM-IR method is given in the Methods section.

As a model system for evaluating photothermal dynamics using the PTM-IR technique, we fabricated a trilayer PMMA/SU-8/PMMA structure in a cleanroom environment. The thickness of each layer was initially measured using profilometry, averaging five points from both the center and the edges of the sample. The mean thicknesses were found to be $L_1= 3.2 \pm 0.1\,\upmu \hbox {m}$ for the top PMMA layer, $L_2-L_1= 1.16 \pm 0.02\,\upmu \hbox {m}$ for the intermediate SU-8 layer, and $L_3-L_2=2.1 \pm 0.1\,\upmu \hbox {m}$ for the bottom PMMA layer on $\hbox {CaF}_2$ substrate.

**Fig. 2:**
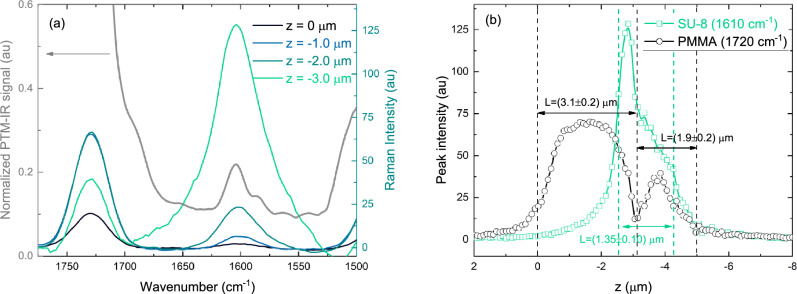
**a** Normalized PTM-IR signal and Raman spectra after baseline correction, showing at $1610\hbox { cm}^{-1}$ and $1720\hbox { cm}^{-1}$, corresponding to the aromatic C=C and ester C=O stretching modes, respectively. **b** Depth profile of the Raman peak intensities at these wavenumbers, used to determine the thicknesses of the individual layers.

To independently verify the layer thicknesses, we performed confocal Raman microscopy on the trilayer sample using a 532 nm excitation and a 100x objective. Figure 2a and b show the Raman spectra and corresponding depth profiles across the layered structure. Two prominent Raman peaks are observed: one at $1610\hbox { cm}^{-1}$, corresponding to the aromatic C=C stretching vibrations, and another at $1720\hbox { cm}^{-1}$ attributed to the C=O vibrational mode of the ester moiety. The depth profiling was carried out by tracking the intensity of these peaks along the *z* axis. Vertical lines indicate the interfaces between layers, from which the layer thicknesses were extracted. The thicknesses of the layers retrieved from the depth profile were found to be around $L_1= 3.1\pm 0.2\,\upmu \hbox {m}$ and $L_3-L_2= 1.9\pm 0.2\,\upmu \hbox {m}$ for the PMMA layers and $L_2-L_1= 1.35\pm 0.15\,\upmu \hbox {m}$ for the SU-8 layer. These results show good agreement with the values obtained from profilometry.

Figure 2a also presents the PTM-IR spectrum of the sample, spanning the range between 1798 and $1488\hbox { cm}^{-1}$. The spectral band centered around $1610\hbox { cm}^{-1}$ is attributed to the C=C stretching vibrations for the SU-8 layer. At wavenumbers above $1700\hbox { cm}^{-1}$, the signal is dominated by absorption from the PMMA layers.

While the PTM-IR spectrum reveals the layer-specific vibrational absorption characteristics, it does not provide information about the temporal dynamics of heat generation and dissipation. To gain further insight into the time-dependent thermal behavior of each material, we performed transient measurements by monitoring the PTM-IR signal over time under modulated laser excitation. These time-domain measurements offer complementary information on thermal rise and relaxation processes within the multilayer structure.

**Fig. 3:**
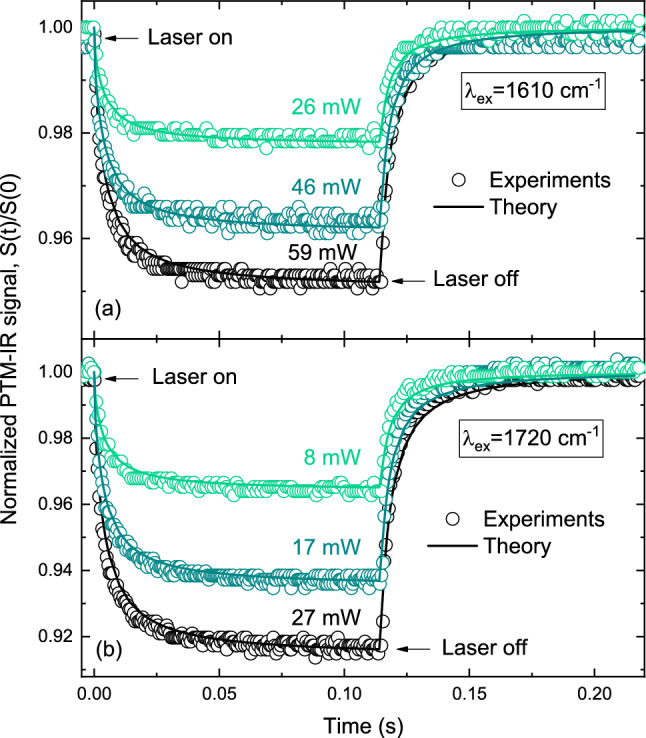
PTM-IR transient signals measured at different excitation powers for two excitation wavenumbers: **a** $1610\hbox { cm}^{-1}$ and **b** $1720\hbox { cm}^{-1}$. Open symbols represent the experimental data, while solid lines correspond to theoretical fits based on Eq. 3.

Figure 3 shows the transient intensity signals acquired under different excitation powers using the PTM-IR method. The sample was selectively excited at the characteristic absorption peaks of each layer–$1610\hbox { cm}^{-1}$ for SU-8 and $1720\hbox { cm}^{-1}$ for PMMA–and the corresponding PTM-IR signal were recorded over time. Each transient trace captures both the heating period (laser on, $115\hbox { ms}$ duration) and the subsequent cooling period (laser off). Note that the signal amplitude is linear with excitation power and that the time-dependent signal is dependent on the thermal diffusivity of the sample.

### Modeling the layered sample

Spectroscopic and transient measurements using the photothermal mirror were performed by exciting the three-layer sample using a tunable continuous excitation laser with a Gaussian transverse spatial distribution ($\hbox {TEM}_{00}$). The laser beam is normally directed to the sample’s surface and propagates along the *z*-axis. Depending on the excitation wavelength, the laser energy is absorbed primarily by one of the layers of the sample and converted to heat. By conduction, heat is transferred to the surrounding layers and substrate, as well as to the fluid (air). The problem is circularly symmetric, and the fields depend only on the normal *z*- and radial *r*-coordinates. The temperature change, $T_{j}(r,z,t)$, in the layers, substrate, and in the surrounding fluid follow separate heat diffusion equations^10,22^

1 $$\begin{aligned} \frac{\partial T_{j}(r,z,t)}{\partial t}- D_{j}\nabla ^{2}T_{j}(r,z,t)=\frac{Q_{0j}}{\rho _j C_{pj}} e^{-2r^{2} /w_{e}^{2}} e^{- \beta _j (z-z_j)}. \end{aligned}$$

$D_{j}=\kappa _{j}/\rho _{j} C_{pj}$ is the thermal diffusivity, $\kappa _{j}$ is the thermal conductivity, $\rho _{j}$ is the mass density, $C_{pj}$ is the specific heat capacity, $\beta _j$ is the Napierian absorption coefficient (in units of $\hbox {cm}^{-1}$) at the excitation wavelength and $w_{e}$ is the radius of the excitation beam in the sample. $z_j$ is the position of the first interface of each layer. The origin of the coordinate system is at the air/film interface. The amplitude of the heat source is $Q_{0j}=(2 P_e \beta _j)/(\pi w_{e}^{2})$, where $P_e$ is the incident laser power.

The time-dependent temperature field is obtained by solving Eq. 1 and then used as the source to calculate the displacement of the sample by solving the thermoelastic equation with the appropriate initial and boundary conditions. The displacement field is given by the solution of^23^

2 $$\left( {\lambda + 2\mu } \right)\nabla ^{2} {\mathbf{u}}_{j} + \left( {\lambda + \mu } \right)\nabla \left( {\nabla \cdot {\mathbf{u}}_{j} } \right) = \gamma \nabla T_{j} (r,z,t) + \rho \frac{{\partial ^{2} {\mathbf{u}}_{j} }}{{\partial t^{2} }},$$

where $\gamma =(3 \lambda +2 \mu )\alpha _{T}$. $\lambda =E \nu /\left[ (1+\nu )(1-2\nu )\right]$ and $\mu =E/2(1+\nu )$ are the Lamé elastic constants, *E* is the Young’s modulus, and $\nu$ is the Poisson’s ratio, $\alpha _{T}$ is coefficient of linear thermal expansion.

The axial surface displacement given by the solution of Eq. 2, $u_z(r,z=0,t)$, is measured by analyzing the on-axis intensity change of the central portion of the probe beam reflected from the sample surface at the far field photodetector. The thermoelastic surface displacement created at the surface acts as the dynamic optical element to the wavefront of the probe beam, increasing or diminishing its amplitude signal passing the pinhole at the detector plane. $u_z(r,0,t)$ produces a phase shift to the reflected portion of the probe beam as $\Phi \left( r,t\right) =(4 \pi /\lambda _p) u_{z} (r,0,t)$, where $\lambda _{p}$ is the probe beam wavelength. The intensity of the reflected probe beam passing the pinhole varies as^24^

3 $$\begin{aligned} S\left( t\right) =\left| \int _{0}^{\infty }\frac{2r}{w_{p}^{2} } \exp \left[ -\left( 1+iV\right) \frac{r^2}{w_{p}^{2} }-i\, \Phi (r,t)\right] \textrm{d}r \right| ^{2} {\hspace{1.0pt}}\,, \end{aligned}$$

where $V=z_1/z_c$, $z_{c}$ is the confocal distance of the probe beam, $z_{1}$ is the distance from the probe beam waist to the sample, and $w_{p}$ is the radius of the probe beam at the sample surface. Eq. 3 can be evaluated numerically using the calculated surface displacement field $u_z(r,0,t)$. Additional phase shift to the probe beam as a consequence of the thermal lens effect created in the surrounding air by heat coupling to the sample, or the influence of the temperature dependence of the physical properties of the samples on the signal, can be safely neglected for the experiments performed in this study.

Equation 3 can be solved numerically by using the Finite Element Analysis (FEA) to calculate Eqs. 1 and 2. The numerical simulations were performed employing realistic boundary conditions by using the software Comsol Multiphysics 5.6. Details are given in the Methods section. The continuous lines in Fig. 3 represent the calculated signal using the optical and thermoelastic physical properties of the layers PMMA, SU-8, air and the substrate $\hbox {CaF}_2$, as given in Table 1. All of the parameters are known for the samples, except the optical absorption coefficients of the layers. The substrate and air absorption coefficient can be neglected in these simulations. From the fitting, the values for the optical absorption coefficients of PMMA and SU-8 were found to be $960 \pm 80\hbox { cm}^{-1}$ at $1610\hbox { cm}^{-1}$ and $1400 \pm 100\hbox { cm}^{-1}$ at $1720\hbox { cm}^{-1}$, respectively. The reported uncertainties reflect propagated errors from sample thickness measurements, obtained through numerical fitting of the experimental data.

**Fig. 4:**
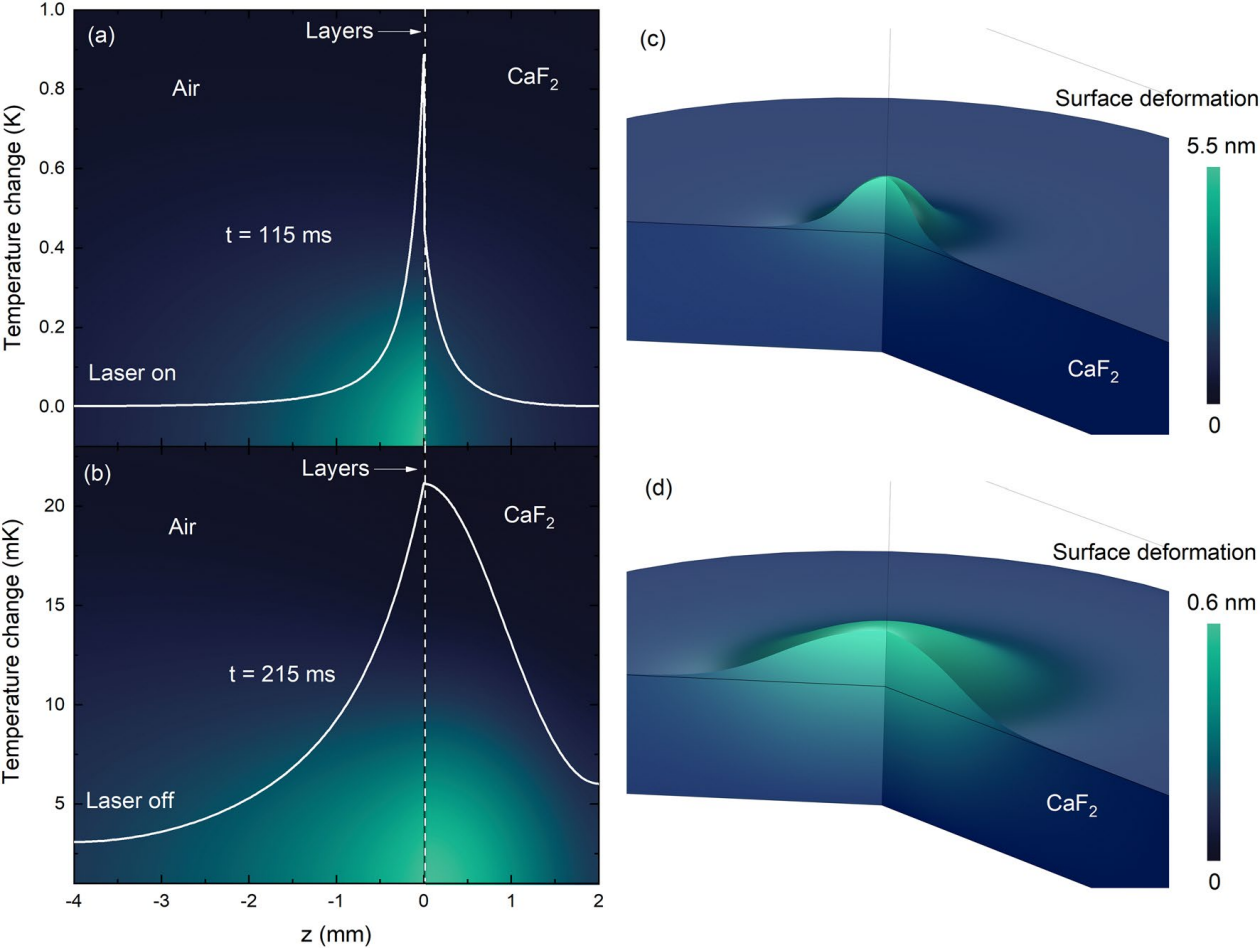
z-dependent **a**–**b** temperature change, Eq. 1, and **c**–**d** thermoelastic displacement, Eq. 2, in the sample at different times for laser on and off. The laser is turned on from $t=0$ to $t=$ $115\hbox { ms}$ and then turned off and the fields are calculated at $t= 115\hbox { ms}$ and $t= 215\hbox { ms}$. The physical properties of the layers, air, and substrate are given in Table 1. These figures were created using COMSOL Multiphysics version 6.0 (https://www.comsol.com/release/6.0) and Origin 2018 (https://www.originlab.com/2018).

With the fitting parameters, the temperature distribution along the *z* axis, $T(r=0,z,t)$, and the thermoelastic surface displacement, $u_z(r=0,z,t)$, within the $\hbox {CaF}_2$ and layers can be calculated. These results are presented in Fig. 4 for two times: during laser excitation at $t= 115\hbox { ms}$ and after the laser is turned off, at $t= 215\hbox { ms}$.

While the FEA simulations provide detailed insight into the full 3D thermal and mechanical behavior of the layered structure, they are computationally intensive and offer limited analytical transparency. Moreover, the observed surface displacement results from the coupled thermoelastic response of both the multilayer stack and the substrate, making it challenging to isolate the contribution of individual layers. To gain a more intuitive understanding of the underlying physical mechanisms and to facilitate efficient parameter exploration, we developed a 1D analytical model. In this framework, closed-form solutions for the temperature distribution and resulting thermoelastic displacement can be obtained under a set of simplifying assumptions.

This simplification is achieved by treating the system in a 1D configuration, which is particularly useful for visualizing the spatial distribution of fields under specific conditions. In the 1D model, the PMMA/SU-8/PMMA trilayer is treated as an effective medium, assuming homogeneous thermal properties across the stack. This approximation is justified by the similar values of density, thermal conductivity, and heat capacity among the individual layers, as summarized in Table 1. Analytical solutions for Eqs. 1 and 2 can then be derived by reducing the original axisymmetric problem to a 1D form, following the approach outlined in^25^

4 $$\begin{aligned} \frac{\partial T_j(z,t)}{\partial t} - D_j\frac{\partial ^2T_j(z,t)}{\partial z^2} = \frac{D_j}{\kappa _j} g_j(z,t). \end{aligned}$$

The subindex *j* denotes the layer materials, where $j =1$ corresponds to PMMA/SU-8/PMMA layers, $j =2$ corresponds to $\hbox {CaF}_2$. The surrounding air is neglected in this analysis. The heating function is defined as $g_j(z,t) = g\cdot \prod (\frac{t}{t_p})\cdot e^{-\beta (z-z_j)}$, where $g_j=\frac{Q_{0j}}{w_e}$. $\prod (\frac{t}{t_p})$ represents the laser pulse function, where $t_p$ is the pulse width.

The physical properties of the three polymer layers are averaged to form an effective medium. The temperature distribution in the multilayer system is determined by the coupled thermal properties of both the polymer film and the substrate. Interface boundary conditions enforce continuity of temperature and heat flux, leading to eigenvalue solutions that reflect this coupling (see Eqs. 11 and 12 in the Methods Section). As demonstrated in Ref. ^10^, the substrate’s thermal properties significantly influence the heat dissipation dynamics within the polymer layer.

In the following, we consider the case where SU-8 is treated as the absorption layer (from $L_1$ to $L_2$), with detailed derivations provided in the Methods section. The temperature solution for the layers is given by

5 $$\begin{aligned} T_1(z,t) = \frac{2g_1}{\kappa _1L_4}\cdot \sum _{n=0}^{\infty }\int _{\tau =0}^{t}e^{-D_{1} \lambda _n^2 (t-\tau ) }\prod (\frac{\tau }{t_p})d\tau \cdot cos\lambda _n z\int _{z'=L_1}^{L_2}cos\lambda _n z'e^{-\beta (z'-L_1)}dz'. \end{aligned}$$

To model the mechanical response of the system, a quasi-static approximation is employed, assuming that thermal loading occurs slowly enough to neglect inertial (acceleration) effects. Specifically, the last term in Eq. 2 is omitted. Under this assumption, the mechanical response is considered to follow the thermal field instantaneously, with no contribution from elastic wave propagation. The 1D thermoelastic equation then simplifies to^26^

6 $$\begin{aligned} \left( \lambda +2 \mu \right) \frac{\partial ^2u_j}{\partial z^2}=\gamma \frac{\partial T_j}{\partial z}, \end{aligned}$$

and is solved following the same procedure described in the Methods section. The net vertical displacement of the surface due to thermal expansion is obtained by integrating the temperature field weighted by the local thermal expansion coefficient and Poisson’s ratio across the entire multilayer structure. As a result,

7 $$\begin{aligned} u_{\textrm{surface}}(t) = \frac{1+v_1}{1-v_1}\alpha _{T_1} \int _0^{L_3} T_1(z',t)dz'+ \frac{1+v_2}{1-v_2}\alpha _{T_2} \int _{L_3}^{L_4} T_2(z',t)dz'. \end{aligned}$$

**Fig. 5:**
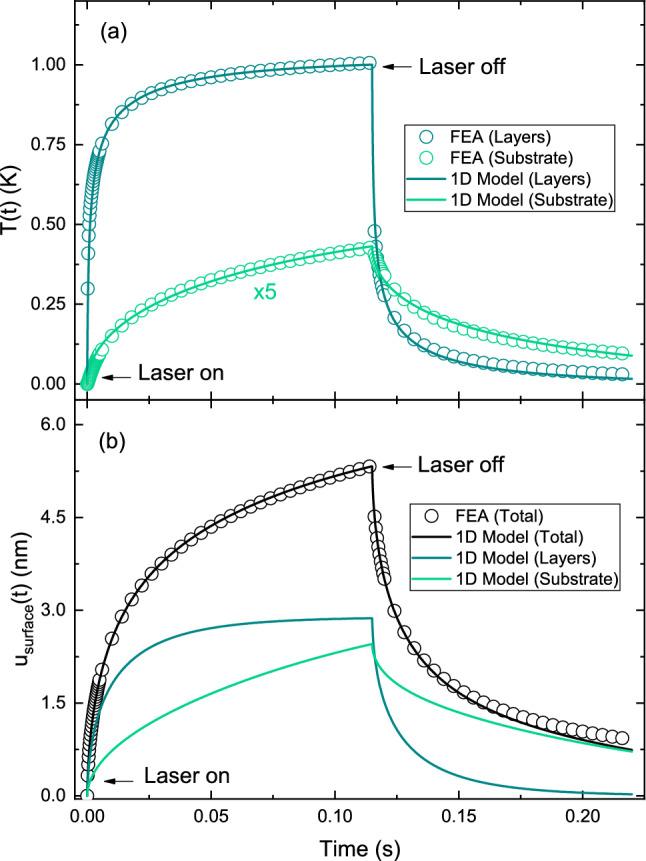
Time-dependent **a** spatially averaged temperature change *T*(*t*) and **b** thermoelastic surface displacement $u_{\textrm{surface}}(t)$ at $r = 0$, obtained from FEA and the 1D analytical model. The temperature for the substrate is scaled by a factor of 5 for visualization clarity. While the substrate temperature near the film is comparable to that of the film, the average film temperature appears  10$\times$ higher due to the substrate average being taken over the full 2 mm thickness, including regions beyond the heated area. Open symbols are the results obtained using FEA at the sample surface, while continuous lines correspond to predictions from the 1D analytical model.

The simplified 1D model provides useful insight into the temperature distribution and surface displacement along the *z* axis within the layers and substrate. Figure 5a presents the time-dependent, spatially averaged temperature change, demonstrating good agreement between the 1D model and the FEA results. Similarly, the thermoelastic displacement at the sample surface ($r=0, z= 0$) closely matches the combined response of the substrate and the three-layer stack as predicted by the 1D model, further validating the underlying assumptions of the analytical approach.

**Fig. 6:**
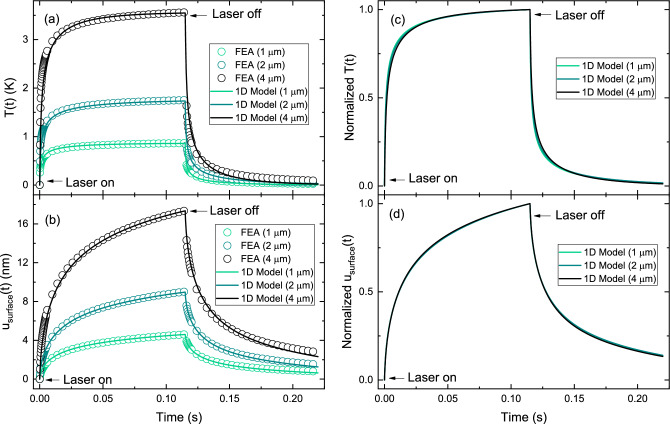
Time-dependent thermal and mechanical response of the trilayer PMMA/SU-8/PMMA sample with varying SU-8 thicknesses: $1\,\upmu \hbox {m}$, $2\,\upmu \hbox {m}$ and $4\,\upmu \hbox {m}$. **a** Spatially averaged surface temperature change *T*(*t*), and **b** surface displacement $u_{\textrm{surface}}(t)$. Open symbols represent FEA results, while solid lines correspond to the 1D model predictions. **c** and **d** Normalized temperature and displacement fields, respectively.

The PTM signal has previously been shown to exhibit a linear dependence on the thickness of single thin films deposited on $\hbox {CaF}_2$ substrates, as reported in Ref.^10^. It is of interest to investigate whether this linearity persists in more complex multilayer systems. To explore this, we examined SU-8 layers of three different thicknesses–$L_2 - L_1=$ $1\,\upmu \hbox {m}$, $2\,\upmu \hbox {m}$ and $4\,\upmu \hbox {m}$–embedded within a trilayer PMMA/SU-8/PMMA configuration. The PMMA layer and substrate thicknesses were kept consistent with previous definitions. The time-dependent spatially averaged temperature change and thermoelastic surface displacement for these cases are shown in Fig.6a and b, respectively. The results clearly indicate that both temperature and displacement signals increase linearly with the thickness of the SU-8 layer.

To further investigate the shape of the temporal response, Fig. 6c and d present the corresponding normalized fields. These plots reveal that while the signal amplitudes vary with thickness, the temporal profiles remain nearly identical.

## Discussion

The PTM-IR method presented here was used to perform spectroscopic and transient analysis of a trilayer PMMA/SU-8/PMMA structure on a $\hbox {CaF}_2$ substrate. To demonstrate its capability as a tool for retrieving optical absorption coefficients, we fabricated the sample in a cleanroom and measured layer thicknesses using profilometry. Complementary confocal Raman microscopy confirmed the layer structure and provide independent thickness estimates, as shown in Fig. 2. Using the measured thicknesses and literature values for material properties (listed in Table 1), we extracted the optical absorption coefficients of SU-8 and PMMA by fitting the PTM-IR data with FEA. The extracted absorption coefficients are $960\hbox { cm}^{-1}$ (SU-8 at an excitation wavenumber of $1610\hbox { cm}^{-1}$ ) and $1400\hbox { cm}^{-1}$ (PMMA at $1720\hbox { cm}^{-1}$).

Figure 4 presents the time evolution of temperature and surface displacement in the layered sample. Both reach their maximum at $t= 115\hbox { ms}$ during laser excitation and gradually relax after the laser is turned off, as shown at $t=$ $215\hbox { ms}$. The mismatch of thermal properties between the layers and substrate leads to distinct dynamic behaviors in temperature and displacement. Laser-induced heating affects the polymer layers, the $\hbox {CaF}_2$ substrate, and the surrounding air, creating a thermal gradient that drives the surface displacement. This results in a transient convex mirror effect that alters the propagation of the reflected probe beam. As the mirror curvature increases, the probe beam diverges at the detector, giving rise to the decaying transient signal observed in Fig. 3.

In addition to extracting absorption coefficients, the PTM-IR signal can also be used to estimate layer thicknesses when the absorption coefficients are known. Both the amplitude and characteristic decay time of the transient signal are sensitive to the thermal and mechanical properties of the sample. As shown in Fig. 5, the temperature reaches a steady-state value more quickly than the surface displacement. The temperature profile is primarily governed by the thin-film layers, whereas the substrate plays a dominant role in determining the displacement response, particularly evident in Fig. 5b.

To examine the effect of SU-8 thickness, we analyzed samples with different layer thicknesses. As shown in Fig. 6a and b, both the temperature change and surface displacement increase linearly with SU-8 thickness. However, the normalized fields in Fig. 6c and d exhibit nearly identical temporal profiles, indicating that the shape of the transient response is mainly independent of the absorbing layer thickness. We can observe that the shape of the normalized transient response is independent of SU-8 layer thickness. This behavior indicates that the system operates in a regime where the overall heat dissipation is dominated by the thermal properties of the entire stack and substrate, rather than just the absorbing layer. The independence of the normalized shape suggests that thermal diffusivity governs the rate of temperature equilibration, but extracting a precise value from this response alone is nontrivial due to coupling between radial and axial diffusion, and contributions from both the film and the substrate.

## Conclusion

In summary, this study demonstrates the effectiveness of photothermal mirror-infrared (PTM-IR) spectroscopy for characterizing thermal and thermoelastic dynamics in multilayer thin-film systems. By combining PTM-IR measurements with finite element simulations and analytical modeling, we accurately retrieved material parameters such as optical absorption coefficients and quantified the time-dependent temperature and surface displacement across a trilayer polymer stack. The 1D analytical model, while computationally efficient, provided clear physical insight into the role of the substrate, layer thicknesses and thermal properties in governing the time-dependent response, validating the observed dynamics. Our results reveal key insights into the temporal decoupling of thermal and mechanical responses, with temperature reaching steady-state significantly earlier than surface displacement. Furthermore, we showed that increasing the thickness of the intermediate SU-8 layer leads to a linear increase in both temperature rise and surface displacement, while preserving the normalized temporal dynamics. These findings position PTM-IR as a robust, non-invasive platform for probing depth-dependent thermal processes in complex heterostructures. This approach holds promise for advancing the design and analysis of functional thin films in emerging applications such as thermal metasurfaces, flexible electronics, and quantum photonic systems.

## Methods

### PTM-IR setup

The excitation laser operates in continuous wave (CW) mode and was provided by a tunable external cavity quantum cascade laser (EC-QCL) (Hedgehog, DRS Daylight Solutions Inc., model 41062-HHG-UT) within the spectral range of 1798 to $1488\hbox { cm}^{-1}$. The beam was modulated at a frequency of $35\hbox { Hz}$ (duty cycle 50%) using a mechanical shutter (Stanford Research Systems, Model SR475) for spectroscopic measurements. For the transient analysis, the beam was modulated at $9\hbox { Hz}$. The excitation beam was split in a 50:50 ratio using a ZnSe beam splitter (Thorlabs, model BSW711). The intensity of the reflected beam was measured with a infrared detector (VIGO Photonics, model LabM-I-10.6) connected to a lock-in amplifier (Zurich Instruments, model MFLI $500\hbox { kHz}$) and used as a reference for the excitation power. The transmitted beam was focused on the position of the sample using a ZnSe lens ($\hbox {OL}_3$) with a focal length f = 0.2 m. A diode-pumped solid-state (DPSS) cw $\hbox {TEM}_{00}$ laser at 633 nm (Coherent, model OBIS 633 nm LX 70 mW) with a power of 35 mW, almost collinear to the excitation beam ($\gamma < 2 ^o$; see Fig. 1), focused by lens $\hbox {OL}_4$ ($\hbox {f} = 0.15\hbox { m}$), was used to probe the periodic surface displacement induced by the modulated excitation beam. The intensity of the center of the probe beam after reflection on the sample surface was maximized by adjusting the mirror $\hbox {M}_4$ and detected by a photodetector (Femto, Model OE-300-SI-10-FST, $200\hbox { MHz}$ bandwidth). The lock-in amplifier demodulates the signal at both the photodetector and power meter using the frequency of the optical shutter as a reference. For each sample, an average of 5 spectra was computed. Each spectrum was recorded with the step and measure tuning mode of the EC-QCL controller with a step size of $1\hbox { cm}^{-1}$, and stabilization time of $335\hbox { ms}$ followed by an acquisition time of $30\hbox { ms}$. An oscilloscope (Rigol, model DS1104) recorded the time-dependent signal averaged over 128 transients for the transient analysis. The radii of the excitation and probe beams in the sample are $350\,\upmu \hbox {m}$ and $800\,\upmu \hbox {m}$, respectively, and the parameter $V=9.3$.

### Confocal Raman microscopy

Confocal Raman microscopy was performed using a *WITec alpha300 RSA* system equipped with a 532 nm diode-pumped solid-state laser as the excitation source. The laser was focused onto the sample using a 100$\times$ objective with a numerical aperture of 0.9. The incident laser power was set to 5 mW to minimize sample heating or damage. The scattered light was collected and analyzed using a 600 grooves/mm grating spectrometer coupled to a thermo-electrically cooled CCD detector. Each spectrum was recorded with an integration time of 1.5 seconds, and 30 spectra were averaged to improve the signal-to-noise ratio. Measurements were carried out at room temperature under ambient conditions. The samples were mounted on an XYZ stepper motor stage for precise spatial positioning. Depth-resolved measurements were performed from $2\,\upmu \hbox {m}$ above the surface to $8\,\upmu \hbox {m}$ below the surface, using a step size of 100 nm.

Data processing, including background subtraction and peak fitting, was performed using custom Python scripts implementing the asymmetrically reweighted penalized least squares (arPLS) algorithm^27^, with a column vector of $N=50$ points and a smoothness parameter $\lambda = 10^5$. Raman peak assignments were made based on literature values for PMMA and SU-8.

### Finite element analysis

The numerical solutions to the heat diffusion and thermoelastic equations were performed using the FEA with realistic boundary conditions imposed by the experimental geometry. The model was built in the 2D axisymmetric geometry using the software Comsol Multiphysics 5.6. The ‘Heat Transfer in Solids’ and ‘Solid Mechanics’ modules were used to obtain the temperature and normal component of the surface displacement in the thin films, air and $\hbox {CaF}_2$ substrate. The values of the thermal, mechanical, and optical properties used for the FEA modeling simulations are shown in the Methods section. Realistic sample geometry was considered, i.e., a substrate with 2 mm thickness and 15 mm radius, with 10 mm of air surrounding the sample. The heat diffusion equation (Eq. 1) is solved for all the domains. The thermoelastic equation (Eq. 2) is solved for the thin films and substrate domain. There are four types of boundaries in the model. One boundary represents the axis of symmetry (at $r=0$), which coincides with the center of the excitation laser beam. The external boundaries are defined as thermal insulation. The thin film-air interface is free and has no loads or constraints. The lateral and back surfaces of the substrate are defined as fixed constraints.

### Green function approach

To derive the analytical solution for the temperature change and resulting surface displacement, we reduce Eq. 1 to a one-dimensional heat conduction problem defined over a finite domain $0\le z \le L_4$. The sample is modeled as a multilayer structure, with the properties and thicknesses of each layer illustrated in Fig. 1. A semi-infinite substrate is truncated at depth *L* for analytical tractability. As a result, the total thickness considered is $L_4 = L_3 + L$.

In the following analysis, the system is divided into two regions: Region 1 ($0\le z \le L_3$), which includes a heat source, and Region 2 ($L_3\le z \le L_4$), where no heat source is present. The corresponding mathematical formulation is given by:

8 $$\begin{aligned} \frac{\partial ^2T_j(z,t)}{\partial z^2} + \frac{g_1(z,t)}{\kappa _1} = \frac{1}{D_j}\frac{\partial T_j(z,t)}{\partial t} \end{aligned}$$

where $T_j(z,t)$ is the temperature profile in each region, and $g_1(z,t)$ represents the heat source term in layers. To solve this, we apply the appropriate boundary and initial conditions for the system.

9 $$\begin{aligned} \begin{aligned}&BC1: \quad \frac{\partial T_1}{\partial z}|_{z=0} = 0 \quad \text {top surface is insulating}\\&BC2: \quad T_1(L_3,t) = T_2(L_3,t) \quad \text {continuity of temperature at interface} \\&BC3: \quad -\kappa _1\frac{\partial T_1}{\partial z}|_{z=L_3} = -\kappa _2\frac{\partial T_2}{\partial z}|_{z=L_3} \quad \text {continuity of heat flux at interface}\\&BC4: \quad \frac{\partial T_2}{\partial z}|_{z=L_4} = 0 \quad \text {bottom surface is insulating}\\&IC: \quad T_1(z, t=0) =T_2(z, t=0) = F(z) \quad \text {initial conditions} \end{aligned} \end{aligned}$$

where $\kappa _1$ and $\kappa _2$ are thermal conductivities in Region 1 and Region 2, respectively.

With these boundary conditions in place, we first solve the homogeneous heat conduction equations in Region 1. This leads to the Green’s function, which expresses the temperature response in Region 1 to a point source at $z'$ and time $\tau$. The Green’s function for this system is derived as:

10 $$\begin{aligned} G(z,t|z',\tau ) = \frac{2}{L_3}\sum _{n=0}^{\infty }e^{-D_{1} \lambda _n^2 (t-\tau ) } cos\lambda _n zcos\lambda _n z' \end{aligned}$$

where $\lambda _n$ are the eigenvalue solutions depending on the boundary conditions at the interfaces between Region 1 and Region 2, as specified by BC2 and BC3. These eigenvalues are found by solving the characteristic equation resulting from the continuity conditions at the interfaces, which leads to the eigenmode solutions given by:

11 $$\begin{aligned} \tan \lambda _n L = -\frac{\kappa _1}{\kappa _2}\tan \lambda _n L_3, \quad \text {n = 1,2,3,..} \end{aligned}$$

These eigenvalue solutions are sufficient to satisfy the boundary conditions at the interfaces between layers and substrate. However, due to the substantial mismatch in material properties between Region 1 and Region 2, the substrate must be treated as a distinct domain with its own eigenmodes. For a sufficiently thick substrate, we assume that within short timescales, the temperature at the bottom surface remains close to its initial value and the temperature change can therefore be approximated as zero. This Dirichlet boundary condition at $z = L_4$ leads to the following eigenvalue solutions in Region 2:

12 $$\begin{aligned} \lambda _m = \frac{(m+1)\pi }{L} \quad \text {with \quad m =0,1,2,3...} \end{aligned}$$

Substitution the Green’s function into the non-homogeneous equation yielding the overall temperature solution in Region 1:

13 $$\begin{aligned} T_1(z,t) = \frac{2g_1}{\kappa _1L_4}\cdot \sum _{n=0}^{\infty }\int _{\tau =0}^{t}e^{-D_{1} \lambda _n^2 (t-\tau ) }\prod (\frac{\tau }{t_p})d\tau \cdot cos\lambda _n z\int _{z'=L_1}^{L_2}cos\lambda _n z'e^{-\beta (z'-L_1)}dz' \end{aligned}$$

where $\Gamma _1(t)$ is the time-dependent function defined as:

14 $$\begin{aligned} \Gamma _1(t)=D_{1}\int _{\tau =0}^{t}e^{-D_{1} \lambda _n^2 (t-\tau ) }\prod (\frac{\tau }{t_p})d\tau = \frac{1}{ \lambda ^2_{n}}\left\{ \begin{array}{rcl}{1-e^{-D_{1} \lambda ^2_{n}t}} & \text{ for }& 0 \le t \le t_p \\ \\ (e^{D_{1} \lambda ^2_{n}t_p}-1)e^{-D_{1} \lambda ^2_{n}t} & \text{ for } & t>t_p \end{array}\right. \end{aligned}$$

The space-dependent function $I_n(z)$ is give by:

15 $$\begin{aligned} I_n(z)&= cos\lambda _n z\int _{z'=L_1}^{L_2}cos\lambda _n z'e^{-\beta (z'-L_1)}dz'\end{aligned}$$

16 $$\begin{aligned}&=cos\lambda _n z\frac{\lambda _n(\sin \lambda _n L_2e^{-\beta (L_2-L_1)} - \sin \lambda _n L_1) +\beta (\cos \lambda _nL_1 - \cos \lambda _n L_2e^{-\beta (L_2-L_1)})}{\lambda _n^2+\beta ^2} \end{aligned}$$

In Region 2, the temperature solution $T_2(z,t)$ is derived using the same approach, applying the BC3 at the interface and BC4 at the bottom surface. The temperature solution in this region is given by:

17 $$\begin{aligned} T_2(z,t) = I_nZ_2(z)\Gamma _2(t) \end{aligned}$$

where the eigenfunction in Region 2 is:

18 $$\begin{aligned} Z_2(z)= C_2\left( \cos \lambda _n L_3\cos \lambda _m (z-L_3) -\frac{\kappa _1}{\kappa _2}\sin \lambda _n L_3\sin \lambda _m (z-L_3)\right) \end{aligned}$$

And time-dependent function $\Gamma _1(t)$

19 $$\begin{aligned} \Gamma _2(t)= \frac{1}{D_{2} \lambda ^2_{n}}\left\{ \begin{array}{rcl}{1-e^{-D_{2} \lambda ^2_{n}t}} & \text{ in }& 0 \le t \le t_p \\ \\ (e^{D_{2} \lambda ^2_{n}t_p}-1)e^{-D_{2} \lambda ^2_{n}t} & \text{ for } & t>t_p \end{array}\right. \end{aligned}$$

The equation for surface displacement is derived from the thermal elasticity theory:

20 $$\begin{aligned} \frac{\partial ^2u_j}{\partial z^2} = \frac{\gamma }{\left( \lambda +2 \mu \right) } \frac{\partial T_j}{\partial z} = \frac{1+v}{1-v}\alpha _{Tj}\frac{\partial T_j}{\partial z} \end{aligned}$$

At a free surface at $z=0$ and $z=L_4$, the stress components normal and tangential to the surface are zero. In 1D (or for normal stress in the z-direction), this simplifies to:

21 $$\begin{aligned} \sigma _{zz} = 0 \quad \text {at the free surface} \end{aligned}$$

The total stress includes both mechanical and thermal contributions:

22 $$\begin{aligned} \sigma _{zz} = (\lambda +2 \mu )\frac{\partial u_j}{\partial z}-\gamma T_j \end{aligned}$$

The solution of the thermal displacement is:

23 $$\begin{aligned} u_j(t) = \frac{1+v_j}{1-v_j}\alpha _{Tj} \int T_j(z,t)dz \end{aligned}$$

where $v_j$ is the Poisson’s ratio. This equation provides the final expression for the surface displacement, which is obtained by integrating the temperature distribution across each layer.

### Physical properties

See Table 1.

**Table 1:** Thermoelastic properties of the samples^28^.

Properties	$\rho (kg/m^3)$	$\kappa (W/mK)$	$C_p (J/kgK)$	$\alpha _T (10^{-6}1/K)$	*E*(*GPa*)	$\nu$
Air	1.18	0.026	1007	–	–	–
$\hbox {CaF}_2$	3180	9.71	854	18.85	75.8	0.26
PMMA	1190	0.19	1420	70	3	0.4
SU-8	1200	0.20	1500	52	3.5	0.22

## Data Availability

The datasets used and/or analysed during the current study are available from the corresponding author on reasonable request.

## References

[1] Agne, M. T., Böger, T., Bernges, T. & Zeier, W. G. Importance of thermal transport for the design of solid-state battery materials. *PRX Energy* **1**, 031002. 10.1103/PRXEnergy.1.031002 (2022).

[2] Rousseau, E. et al. Radiative heat transfer at the nanoscale. *Nat. Photonics* **3**, 514–517. 10.1038/nphoton.2009.144 (2009).

[3] Wang, M. et al. High throughput nanoimaging of thermal conductivity and interfacial thermal conductance. *Nano Lett.* **22**, 4325–4332. 10.1021/acs.nanolett.2c00337 (2022). 35579622 10.1021/acs.nanolett.2c00337

[4] Jiang, P., Qian, X. & Yang, R. Tutorial: time-domain thermoreflectance (TDTR) for thermal property characterization of bulk and thin film materials. *J. Appl. Phys.* **124**, 161103. 10.1063/1.5046944 (2018).

[5] Chen, G. *Nanoscale Energy Transport and Conversion: A Parallel Treatment of Electrons, Molecules, Phonons, and Photons* (MIT-Pappalardo series in mechanical engineering (Oxford University Press, Oxford, 2005).

[6] Mazza, G., Gandolfi, M., Capone, M., Banfi, F. & Giannetti, C. Thermal dynamics and electronic temperature waves in layered correlated materials. *Nat. Commun.* **12**, 6904. 10.1038/s41467-021-27081-2 (2021). 34824212 10.1038/s41467-021-27081-2 PMC8616949

[7] Schmidt, A. J., Chen, X. & Chen, G. Pulse accumulation, radial heat conduction, and anisotropic thermal conductivity in pump-probe transient thermoreflectance. *Rev. Sci. Instrum.* **79**, 114902. 10.1063/1.3006335 (2008). 19045906 10.1063/1.3006335

[8] Cahill, D. G. Thermal conductivity measurement from 30 to 750 K: the 3w method. *Rev. Sci. Instrum.* **61**, 802–808. 10.1063/1.1141498 (1990).

[9] Li, N. et al. Colloquium: Phononics—manipulating heat flow with electronic analogs and beyond. *Rev. Mod. Phys.* **84**, 1045–1066. 10.1103/RevModPhys.84.1045 (2012).

[10] Yilmaz, U. et al. Novel insights into nanoscale surface displacement detection in polystyrene thin films using photothermal mirror- and atomic force microscopy-mid-IR spectroscopy. *RSC Adv.* **15**, 9243–9253. 10.1039/D5RA00555H (2025). 40144002 10.1039/d5ra00555h PMC11938214

[11] Lee, S., Yeom, B., Kim, Y. & Cho, J. Layer-by-layer assembly for ultrathin energy-harvesting films: piezoelectric and triboelectric nanocomposite films. *Nano Energy* **56**, 1–15. 10.1016/j.nanoen.2018.11.024 (2019).

[12] Garlisi, C. et al. Multilayer thin film structures for multifunctional glass: self-cleaning, antireflective and energy-saving properties. *Appl. Energy* **264**, 114697. 10.1016/j.apenergy.2020.114697 (2020).

[13] Jung, Y. S. et al. Balanced performance enhancements of a-InGaZnO thin film transistors by using all-amorphous dielectric multilayers sandwiching high-k . *Adv. Electron. Mater.* **5**, 1900322. 10.1002/aelm.201900322 (2019).

[14] Rombaut, J., Martínez, S., Matera, U. M., Mazumder, P. & Pruneri, V. Antireflective multilayer surface with self-cleaning subwavelength structures. *ACS Photonics* **8**, 894–900. 10.1021/acsphotonics.0c01909 (2021).

[15] Paul, D. D. Optical metamaterials: fundamentals and applications. *Phys. Today* **63**, 57–58. 10.1063/1.3490504 (2010).

[16] Joannopoulos, J. D., Winn, J. N. & Johnson, S. G. *Photonic Crystals: Molding the Flow of Light* 2nd edn. (Princeton University Press, Princeton, NJ, 2011).

[17] Jana, R., Hajra, S., Rajaitha, P. M., Mistewicz, K. & Kim, H. J. Recent advances in multifunctional materials for gas sensing applications. *J. Environ. Chem. Eng.* **10**, 108543. 10.1016/j.jece.2022.108543 (2022).

[18] Yu, N. & Capasso, F. Flat optics with designer metasurfaces. *Nat. Mater.* **13**, 139–150. 10.1038/nmat3839 (2014). 24452357 10.1038/nmat3839

[19] Dan, A., Barshilia, H. C., Chattopadhyay, K. & Basu, B. Solar energy absorption mediated by surface plasma polaritons in spectrally selective dielectric-metal-dielectric coatings: A critical review. *Renew. Sustain. Energy Rev.* **79**, 1050–1077. 10.1016/j.rser.2017.05.062 (2017).

[20] Gurevich, Y., Lashkevich, I. & Gonzalez De La Cruz, G. Effective thermal parameters of layered films: an application to pulsed photothermal techniques. *Int. J. Heat Mass Transf.* **52**, 4302–4307. 10.1016/j.ijheatmasstransfer.2009.03.068 (2009).

[21] Zhang, Y. et al. An analytical model of label-free nanoscale chemical imaging reveals avenues toward improved spatial resolution and sensitivity. *Proc. Natl. Acad. Sci.* **122**, e2403079122. 10.1073/pnas.2403079122 (2025). 39854236 10.1073/pnas.2403079122 PMC11789012

[22] Lukasievicz, G. V. B. et al. A theoretical and experimental study of time-resolved thermal mirror with non-absorbing heat-coupling fluids. *Appl. Spectrosc.* **66**, 1461–1467. 10.1366/12-06743 (2012). 23231909 10.1366/12-06743

[23] Nowacki, W. *Thermoelasticity* 2nd edn. (Pergamon Press; PWN-Polish Scientific Publishers, Oxford, Warszawa, New York, 1986).

[24] Astrath, N. G. C. et al. Unveiling bulk and surface radiation forces in a dielectric liquid. *Light Sci. Appl.* **11**, 103. 10.1038/s41377-022-00788-7 (2022). 35443703 10.1038/s41377-022-00788-7 PMC9021243

[25] Hahn, D. W. & Özisik, M. N. *Heat Conduction* 3rd edn. (Wiley, Hoboken, NJ, 2012).

[26] He, T. & Guo, Y. A one-dimensional thermoelastic problem due to a moving heat source under fractional order theory of thermoelasticity. *Adv. Mater. Sci. Eng.* **1–9**, 2014. 10.1155/2014/510205 (2014).

[27] Baek, S.-J., Park, A., Ahn, Y.-J. & Choo, J. Baseline correction using asymmetrically reweighted penalized least squares smoothing. *The Analyst* **140**, 250–257. 10.1039/C4AN01061B (2015). 25382860 10.1039/c4an01061b

[28] Lide, D. R. *CRC Handbook of Chemistry and Physics* 97th edn. (Taylor & Francis Group, Boca Raton, FL, 2017).

